# Reality monitoring impairment in schizophrenia reflects specific prefrontal cortex dysfunction

**DOI:** 10.1016/j.nicl.2017.01.028

**Published:** 2017-01-25

**Authors:** Jane R. Garrison, Emilio Fernandez-Egea, Rashid Zaman, Mark Agius, Jon S. Simons

**Affiliations:** a Department of Psychology, University of Cambridge, UK; b Behavioural and Clinical Neuroscience Institute, University of Cambridge, UK; c Department of Psychiatry, University of Cambridge School of Clinical Medicine, UK; d Cambridgeshire and Peterborough NHS Foundation Trust, UK; e East London Foundation Trust, UK

**Keywords:** Schizophrenia, Prefrontal cortex, Cognitive dysfunction, fMRI, Reality monitoring, Working memory

## Abstract

Reality monitoring impairment is often reported in schizophrenia but the neural basis of this deficit is poorly understood. Difficulties with reality monitoring could be attributable to the same pattern of neural dysfunction as other cognitive deficits that characterize schizophrenia, or might instead represent a separable and dissociable impairment. This question was addressed through direct comparison of behavioral performance and neural activity associated with reality monitoring and working memory in patients with schizophrenia and matched healthy controls. Participants performed a word-pair reality monitoring task and a Sternberg working memory task while undergoing fMRI scanning. Distinct behavioral deficits were observed in the patients during performance of each task, which were associated with separable task- and region-specific dysfunction in the medial anterior prefrontal cortex for reality monitoring and dorsolateral prefrontal cortex for working memory. The results suggest that reality monitoring impairment is a distinct neurocognitive deficit in schizophrenia. The findings are consistent with the presence of a range of dissociable cognitive deficits in schizophrenia which may be associated with variable functional and structural dysconnectivity in underlying processing networks.

## Highlights

• We examined reality monitoring and working memory in patients with schizophrenia.
• Distinct behavioral deficits observed during performance of each task
• Separable task- and region-specific dysfunction in distinct prefrontal cortex regions
• Reality monitoring appears to be a distinct neurocognitive deficit in schizophrenia.

## 1 Introduction

Reality monitoring is the ability to discriminate between internally and externally generated information (Johnson and Raye, 1981), typically tested using source memory paradigms involving the recollection of whether or not information was generated by participants themselves. Numerous studies have observed reality monitoring impairment in patients with schizophrenia compared to healthy controls, often with evidence of a greater reduction than in other aspects of long-term memory (Fisher et al., 2008, Keefe et al., 2002, Vinogradov et al., 1997, Vinogradov et al., 2008).

In healthy individuals, neuroimaging studies have repeatedly observed activity during reality monitoring performance in the medial anterior prefrontal cortex (PFC) (Simons et al., 2006, Simons et al., 2008, Vinogradov et al., 2006). However, despite numerous reports of reality monitoring impairment in schizophrenia, and its possible link with hallucinations (Bentall, 1990, Brookwell et al., 2013), there has been little research to determine the neural basis of this patient deficit. Two studies have investigated the neural correlates of reality monitoring impairment in schizophrenia, reporting reductions in medial anterior PFC activity (BA 10) in patients compared with controls during source memory retrieval of self-generated (imagined) items compared with those that had been externally perceived (Subramaniam et al., 2012, Vinogradov et al., 2008). However, it is unclear whether the reality monitoring impairment observed in patients with schizophrenia is associated with the same pattern of underlying neural dysfunction as other cognitive deficits that characterize the disorder, or whether instead it represents a separable and dissociable deficiency.

Cognitive deficits are a stable and enduring characteristic of schizophrenia (Barch and Keefe, 2010, Keefe and Fenton, 2007), which have a significant effect on day-to-day functioning (Green et al., 2000, Heaton et al., 2001, Reichenberg and Harvey, 2007). Meta-analyses confirm that patients perform significantly below control subjects across a wide variety of cognitive domains, including visual and verbal episodic memory, attention, problem solving, working memory, and social cognition (Heinrichs and Zakzanis, 1998, Keefe and Fenton, 2007, Mothersill et al., 2014, Nuechterlein et al., 2004, Schaefer et al., 2013). Notable across these studies are consistent reports of reduced activation in patients within the PFC across different cognitive tasks. The dorsolateral PFC exhibits perhaps the most consistent dysfunction across studies, but there is little commonality in other brain regions affected, and no evidence for a deficit in a single localized brain region that can explain all of the group differences in task performance (*i.e.*, no ‘smoking gun’; Libby and Ragland, 2012, p255).

Instead, the neural basis of these cognitive impairments is thought to reflect structural or functional dysconnectivity across PFC-mediated brain networks (Andreasen et al., 1998, van den Heuvel and Fornito, 2014). However, it remains unresolved whether these deficits reflect an underlying broad and generalized impairment (Gold and Dickinson, 2013, Hill et al., 2004, Palmer et al., 2010) as might be explained by a global brain-wide disturbance in network coordination, or instead are better understood as domain specific, reflecting distinct patterns of dysconnectivity across underlying cognitive networks, possibly combined with localized cortical dysfunction (Green et al., 2013, Reichenberg and Harvey, 2007, Savla et al., 2012).

Despite the many neuroimaging studies that have compared brain activity during cognitive task performance in patients with schizophrenia and healthy controls, few have assessed group differences across two or more tasks in the same patient and control participants, enabling a comparison of neural dysfunction across different cognitive domains. Such a within-subjects design was implemented in the current study to address the hypothesis that reality monitoring represents a dissociable neurocognitive deficit in schizophrenia. Behavioral performance and neural activity associated with reality monitoring was compared with that from working memory, in a sample of twenty patients with schizophrenia and twenty healthy controls subjects.

Working memory was chosen as a comparator domain to reality monitoring as it is the best characterized of the cognitive deficits in schizophrenia, often one of the most severely and consistently affected cognitive domains in the disorder (Goldman-Rakic, 1992, Weinberger et al., 1986). Patients have been reported to exhibit deficits across a wide range of working memory tasks, particularly those focusing on information maintenance and updating, and resistance to interference (Aleman et al., 1999, Lee and Park, 2005, Potkin et al., 2009a, Potkin et al., 2009b). Neuroimaging studies involving healthy participants have observed working memory related activity in frontal, parietal, and temporal regions (Owen et al., 2005), and particularly within the dorsolateral PFC (D'Esposito et al., 1999, Manoach, 2003, Rypma and D'Esposito, 1999, Veltman et al., 2003). Dysfunction in this region of the PFC has been most widely associated with the working memory deficits observed in schizophrenia (Goldman-Rakic, 1992), but this dysfunction is not typically reflected in a consistent pattern of hypo- or hyper-activation, instead manifesting in either direction depending on task demands (Manoach, 2003). For instance, although fewer resources are needed at low than high working memory loads in controls, dysfunction in the working memory network in patients with schizophrenia may lead to greater relative dorsolateral PFC activity. As memory load increases, there may be a point of crossover as task difficulty exceeds network capacity for patients but not controls, at which point patients may show reduced activity in the dorsolateral PFC compared with controls.

The present study investigated whether disruption in the medial anterior PFC in patients with schizophrenia during reality monitoring is directly tied to the dysfunction observed in the dorsolateral PFC during working memory, as might be explained by a broad level of global network disruption, or whether it instead reflects a separable functionally-distinct source of impairment explained by domain-specific network dysconnectivity, or by localized PFC dysfunction. We predicted a finding of region and task specificity, based on previous between-study behavioral evidence (*e.g.* see Green et al., 2013), which would support a dissociation hypothesis that reality monitoring impairment in schizophrenia represents a distinct neurocognitive deficit.

## 2 Methods and materials

### 2.1 Participants

Participants comprised 20 patients who met the DSM-V criteria for schizophrenia, as diagnosed by their clinicians and verified using the Mini International Neuropsychiatric Interview (Sheehan et al., 1998), and 20 matched healthy control individuals. All participants were native English speakers who had lived in the UK their whole lives. Written informed consent was obtained from participants in a manner approved by the UK National Research Ethics Service.

To optimize the fMRI analysis, within-group variability was minimized by selecting patients who were clinically stable, high functioning, and able to meet the cognitive and psychological demands of the experiment. The patient and control groups were matched on age, gender, handedness, and verbal IQ (Table 1). Patients exhibited the characteristic deficit in fluid IQ compared with the control participants. All patients were receiving antipsychotic medication but none were on drug regimens that included typical antipsychotics, anticholinergics or benzodiazepines. Participants were screened using the Mini International Neuropsychiatric Interview (Sheehan et al., 1998) to ensure no additional current or previous neurological disorder. Data from the first experimental block for one patient was excluded due to anxiety causing adverse movement in the scanner during that block (*i.e.* translation of > 3 mm, rotation of > 4°).

**Table 1:** Clinical and demographic characteristics.

Characteristic	Control subjects		Patients		Significance	
	Mean	SD	Mean	SD	t/Χ^2^	p
Age (years)	33.4	8.0	36.3	7.4	1.2	0.230
Gender (% male)	90.0		90.0		0.0	1.000
Handedness (% right)	100.0		100.0		0.0	1.000
IQ Verbal^a^	113.4	6.3	110.4	7.1	1.4	0.157
IQ Fluid^b^	112.4	14.4	99.2	15.8	2.8	0.009
PANSS^c^ score - positive symptoms			14.9	4.5		
PANSS^c^ score - negative symptoms			14.1	6.1		
Time since diagnosis (years)			13.6	5.4		
Time on medication (years)			12.4	4.7		

a Verbal IQ measured using the National Adult Reading Test (Nelson, 1982).

b Fluid IQ measured using Raven's Advanced Progressive Matrices (Hamel and Schmittmann, 2006).

c Positive and Negative Symptoms Scale (Kay et al., 1987).

### 2.2 Design

Participants performed three tasks established in the previous literature to assess reality monitoring (Simons et al., 2006, Simons et al., 2008), working memory (Potkin et al., 2009a, Potkin et al., 2009b, Sternberg, 1966), and as a control condition, perceptual motor function (Gilbert et al., 2010, Simons et al., 2006).

#### 2.2.1 Reality monitoring

**Fig. 1:**
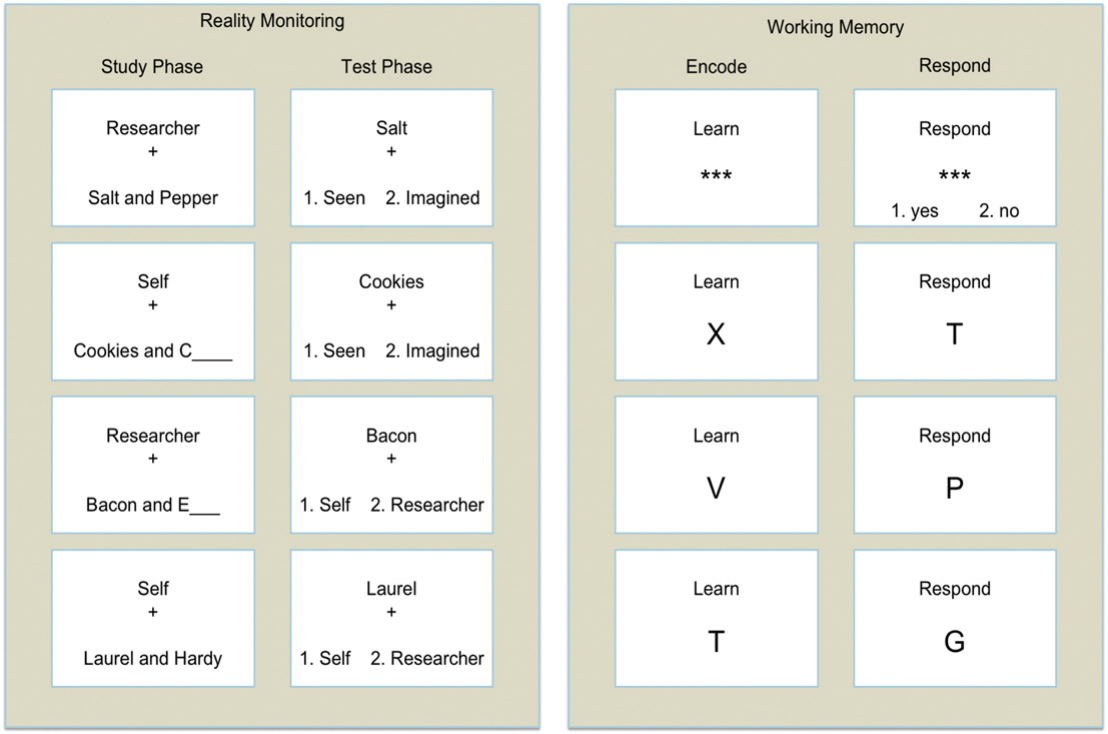
Stimuli used in the reality monitoring and working memory tasks. The top panel shows stimuli used in the study and test phases of the reality monitoring task which employed a 2 × 2 design involving either the subject of researcher speaking aloud the stimuli, which were presented either complete (perceived) or incomplete (the second word to be imagined). The bottom panel shows stimuli used for load-three of the working memory task, the respond phase shows only three out of fourteen trials undertaken, one with a target and two with foils.

Stimuli for the reality monitoring task consisted of 144 well-known word-pairs (*e.g.* ‘Bacon and Eggs’). The task comprised six blocks of a study and test phase, with 24 word-pair stimuli in each (six word-pairs presented in four combinations of Self/Researcher × Perceived/Imagined conditions; Fig. 1). Each study trial commenced with a screen indicating whether the subject or researcher should read aloud the word-pair. The word-pair was then shown, either complete (‘perceived’ trials) or with only the first letter of the second word provided such that the second word needed to be self-generated (‘imagined’ trials). In both cases the subject or researcher then had 2.5 s to read aloud the entire word-pair, completing the word-pair as necessary for imagined trials. Each study phase was followed by its corresponding test phase, half the trials of which tested each of the different reality monitoring conditions. Each half of the test phase commenced with a question screen indicating the condition being tested, “*Was the accompanying word Seen or Imagined?*” or “*Was the accompanying word said by Self or Researcher?*” Each test phase trial involved presentation of the first word from one of the studied word-pairs together with the instruction to provide the appropriate response. Participants had 4.5 s to make their response on a button box, using the first two fingers of their right hand.

The order of presentation of reality monitoring conditions in the test phase alternated across the six full blocks of the task and was counterbalanced across participants. The word-pairs assigned to the perceived/imagined and self/researcher conditions were also counterbalanced across participants, and the order of presentation of word-pairs was pseudo-randomized to ensure no run of more than three items of the same condition in any study or test phase.

#### 2.2.2 Working memory task

A version of the Sternberg Item Recognition Paradigm (SIRP) was used with stimulus loads and timings matching those used in the Functional Imaging Biomedical Informatics Research Network Consortium study into working memory in schizophrenia (Potkin et al., 2009a, Potkin et al., 2009b, Wible et al., 2009), see Fig. 1.

The SIRP task was administered over six scanned blocks, with three working memory loads included in each block. Each level comprised an encode phase, in which participants were instructed to learn one, three, or five stimuli which were presented sequentially as individual randomly selected letters, for 1.1 s each. The encode phase was followed by a respond phase, in which a response was given on a button box, using the first two fingers of the right hand, as to whether a probe stimulus had been included in the learned list. Fourteen probes were used for each level of the task, seven of which were learned targets, and seven non-studied foils. Participants were given 1.1 s to make their response, and a jitter was introduced to the inter-trial interval in both encode and probe phases to maximize design efficiency and ensure participants paid attention to the screen. The working memory block was completed when all three load conditions had been undertaken. The order of presentation of loads was counterbalanced between participants and across blocks, and new randomly selected stimuli were used for each block of the task.

#### 2.2.3 Perceptual motor baseline task

Participants made left and right key presses using the first two fingers of the right hand alternately to make a row of nine ‘X's flip as quickly as possible between a horizontal and vertical orientation. The stimulus was immediately removed from the screen after each key press, followed by a random delay (between 300 and 700 ms) to induce participants to pay attention to the stimuli.

### 2.3 Procedure

**Fig. 2:**
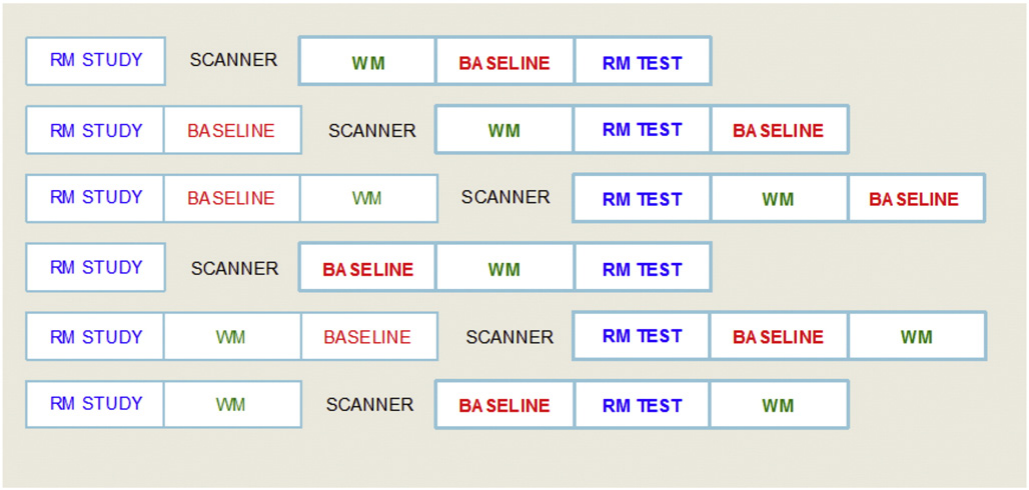
Task order and functional run structure. The order of presentation of the three experimental tasks is shown for each of the six run orders, through which participants cycled. Task order was counterbalanced between participants by starting each participant with a different run order. RM = reality monitoring task, WM = working memory task, BASELINE = perceptual motor baseline task, SCANNER = scanner switch on. Scanned versions of the tasks are shown in bold text. Functional runs lasted from 482 to 700 s but scanning time was fixed at 370 s.

The three tasks were administered over six separate functional scanner runs, which were acquired consecutively during a single visit. Each run comprised one scanned block of each of the three tasks, presented in varying order over the six scans with the run order counterbalanced between participants by starting each participant with a different run order and progressing through the sequence (see Fig. 2). Each run commenced with a spoken reality monitoring study phase, which was not scanned to avoid the disruption of scanner noise and of head movement associated with speech. In four of the six runs, additional versions of the working memory and perceptual motor tasks were administered before the scanner was switched on to equate the time (233 s) and cognitive demands between the study and test phases of the reality monitoring task across counterbalancing orders. The data from these additional versions of the tasks was not analyzed. Each of the six functional runs thus contained a single scanned block of each of the three tasks of interest, occurring in counterbalanced order.

### 2.4 Imaging acquisition and data analysis

A 3 T Siemens Trio system was used to acquire structural and echo-planar functional images (TR 2140 ms, TE 30 ms, 36 sequential axial slices oriented 10–20° to the anterior commissure–posterior commissure transverse plane, 2 mm thickness, 1 mm interslice skip, 3 mm × 3 mm in-plane resolution, 64 × 64 pixels, 78° flip angle, 6 functional runs each of 170 volume acquisitions). To correct for distortion (Hutton et al., 2002), field maps were acquired (TE = 5.19 and 7.65 ms, TR = 400 ms, matrix size = 64 × 64) using 32 slices covering the whole head (voxel size 3 mm × 3 mm × 3 mm). fMRI data were analyzed using SPM8 software (http://www.fil.ion.ucl.ac.uk/spm). The first five volumes from each functional run were discarded to allow for T1 equilibration. The remaining functional volumes were spatially realigned to the first image of the first series, and distortion corrections were applied based on the field maps using the unwarp routines in SPM (Andersson et al., 2001, Hutton et al., 2002). Thereafter, volumes were normalised against the MNI reference brain using tri-linear interpolation, and smoothed with an isotropic 8 mm full-width half-maximum Gaussian kernel.

The volumes acquired during the six scan runs were treated as separate time series. For each run, separate regressors coded for trial onsets for correct judgments for the self, researcher, perceived and imagined conditions in the reality monitoring task, correct responses for each load of the working memory task, and for responses to the perceptual motor baseline task. In each case, these were generated with delta functions corresponding to trial onsets convolved with a canonical hemodynamic response function. These regressors, together with a single regressor representing onsets for incorrect trials from all tasks, and six regressors coding movement parameters, comprised the full model for each run. The data and model were high-pass filtered to a cut off of 1/128 Hz.

Contrasts of interest were performed on individual subject data using the following contrasts between correct response regressors: reality monitoring > perceptual motor baseline task and working memory load 5 > load 3. These contrasts were chosen to maximize power in terms of the number of trials available for the reality monitoring condition, and to maximize the difference in expected signal change in the dorsolateral PFC between groups for the working memory task, based on earlier findings using the same version of the SIRP task, (see Manoach, 2003). Second-level one-sample *t*-tests were performed on the combined individual results to produce random-effects group analyses separately for the healthy control group and for the schizophrenia group. Second-level two-sample *t*-tests were then performed on the combined individual results to enable random-effects between-group analyses.

To test the *a priori* hypothesis that the main contrasts of interest would reveal differences in distinct regions of the PFC, small volume corrections (SVCs) for multiple comparisons were conducted on the whole-brain group-level *t*-tests with a familywise-error (FWE) corrected voxel-wise height threshold of p < 0.05. Regions of interest each comprised an 8 mm radius sphere centered on *a priori* coordinates derived from previous studies carried out on healthy adult participants as follows:

Reality Monitoring: mean of the coordinates of peak activity in left (−16, 56, 14) and right (20, 56, 9) hemisphere medial anterior PFC from eight source memory studies testing recollection of self or other action, or perceived or self-generated stimulus generation (Simons et al., 2005a, Simons et al., 2005b, Simons et al., 2006, Simons et al., 2008, Subramaniam et al., 2012, Turner et al., 2008, Vinogradov et al., 2006, Vinogradov et al., 2008); Working Memory: mean of the coordinates of peak activity in left (−45, 25, 25) and right (43, 38, 18) hemisphere dorsolateral PFC from four previous working memory studies which utilized load related contrasts for analysis of the SIRP task (Altamura et al., 2007, Bunge et al., 2001, Rypma et al., 1999, Veltman et al., 2003). Comparison of activity in each of these regions for the two tasks was undertaken by extracting the percentage signal change for each subject within each voxel, then comparing the mean values using a repeated measures Anova, with Greenhouse-Geisser correction used where necessary to correct for violations of sphericity.

To further explore the nature of the activation associated with the two tasks in the different groups, activations outside the regions of interest were reported if they exceeded a FWE whole-brain corrected voxel-wise height threshold of p < 0.05.

## 3 Results

### 3.1 Behavioral results

**Fig. 3:**
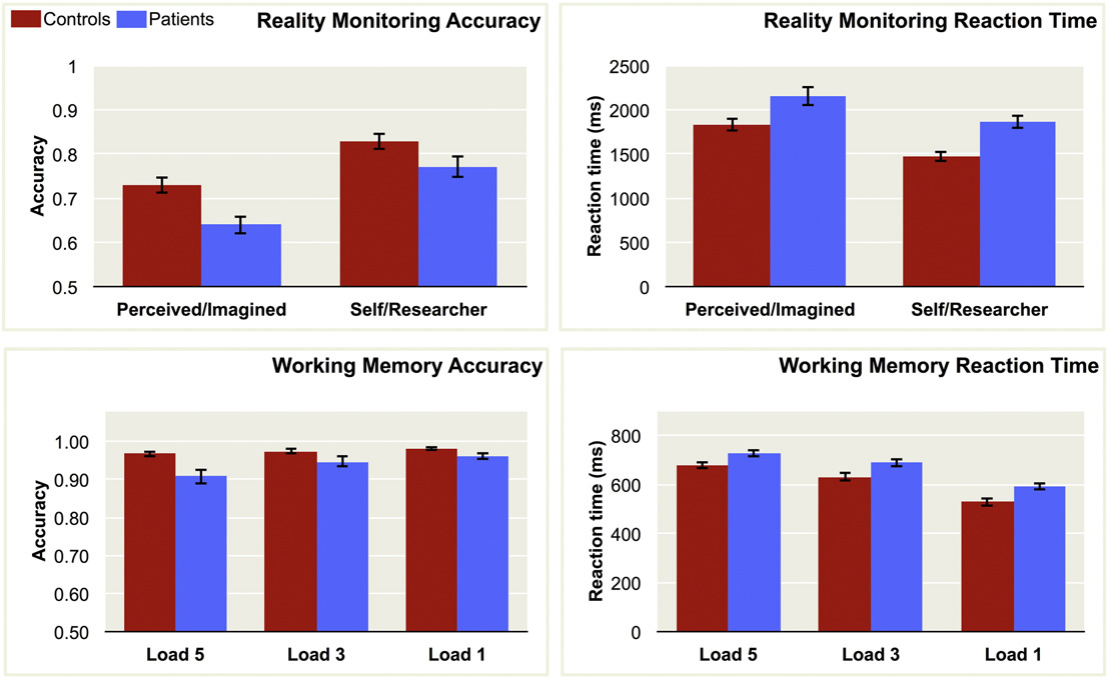
Reality monitoring and working memory task performance. Error bars for all charts represent standard error.

Reality monitoring and working memory accuracy were calculated as number of correct responses made as a percentage of total responses. Patients with schizophrenia were significantly less accurate than controls on both the reality monitoring, F(1,38) = 8.058, p = 0.007, η_p_^2^ = 0.175, and working memory tasks, F(1,38) = 9.985, p = 0.003, η_p_^2^ = 0.208 (Fig. 3). There was a main effect of condition for reality monitoring, with the Self/Researcher question associated with better performance than Perceived/Imagined, F(1,38) = 141.059, p < 0.001, η_p_^2^ = 0.788, and no significant interaction between group and reality monitoring condition, F(1,138) = 2.707, p = 0.108, η_p_^2^ = 0.066. In the working memory task, accuracy for both groups reduced as load increased, F(1,38) = 9.415, p < 0.001, η_p_^2^ = 0.199, with a significant interaction between group and load, F(1,38) = 3.684, p = 0.030, η_p_^2^ = 0.088, indicating that the patients exhibited larger deficits in working memory performance at high loads relative to low loads. There were no significant correlations between reality monitoring accuracy (averaged across conditions) and working memory accuracy (averaged across the three load levels), for either the patients, r = 0.186, p = 0.432, or controls: r = −0.057, p = 0.812, suggesting that the reality monitoring and working memory tasks may have drawn on largely unrelated cognitive processes. There were significant correlations between accuracy on the Self/Experimenter and Perceived/Imagined reality monitoring conditions, in both controls, r = 0.690, p = 0.001, and patients, r = 0.841, p < 0.001, consistent with the notion that similar cognitive processes underlie both forms of reality monitoring.

Patients were slower than controls on both the reality monitoring, F(1,38) = 11.966, p = 0.001, η_p_^2^ = 0.239, and working memory tasks, F(1,38) = 9.971, p = 0.003, η_p_^2^ = 0.208. Self/Researcher responses were made more quickly than Perceived/Imagined responses, F(1,38) = 128.108, p < 0.001 η_p_^2^ = 0.771, with no significant interaction between group and reality monitoring condition, F(1,38) = 1.502, p = 0.228, η_p_^2^ = 0.038. On the working memory task, responses to higher loads were made more slowly for both groups, F(1,38) = 318.695, p < 0.001, η_p_^2^ = 0.893, with no significant interaction between group and load, F(1,38) = 1.109, p = 0.335, η_p_^2^ = 0.028.

### 3.2 Neuroimaging results

**Fig. 4:**
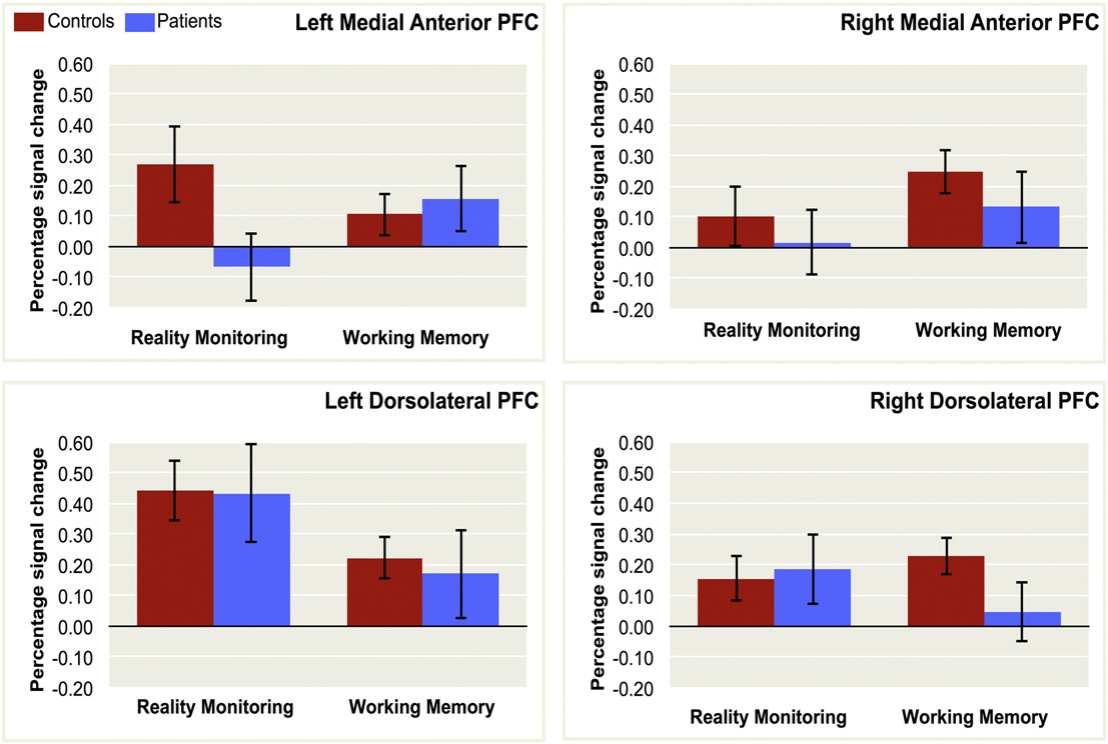
Neuroimaging results – group, region and task level dissociations. Percentage signal change for patients and controls in the reality monitoring and working memory contrasts, within the *a priori* left medial anterior PFC voxel: x = −16, y = 56, z = 14; right medial anterior PFC voxel: x = 20, y = 56, z = 9; left dorsolateral PFC voxel: x = −45, y = 25, z = 25; and right dorsolateral PFC voxel: x = 43, y = 38, z = 18, derived from previous studies.

To investigate whether activity in the medial anterior PFC and dorsolateral PFC regions of interest differed across reality monitoring and working memory, the percentage signal change for each subject and for each contrast was extracted from the left and right medial anterior PFC, and the left and right dorsolateral PFC, *a priori* voxels derived from previous studies (Fig. 4).

A repeated measures ANOVA was conducted with the factors of group (patients and controls), task (reality monitoring and working memory), and region (left and right medial anterior PFC, left and right dorsolateral PFC). This analysis revealed no significant main effects on activity of group, F(1,38) = 1.053, p = 0.311, η_p_^2^ = 0.027, or task, F(1,38) = 0.117, p = 0.735, η_p_^2^ = 0.003, but there was a main effect of region, F(1,38) = 6.507, p = 0.001, η_p_^2^ = 0.146. There were no significant two-way interactions involving the factor of group, F(1,38) < 0.389, p > 0.761, η_p_^2^ < 0.010, but there was a significant interaction between task and region, F(1,38) = 0.784, p = 0.009, η_p_^2^ < 0.120. Crucially there was also a significant three-way interaction between group, task, and region, F(1,38) = 3.423, p = 0.041, η_p_^2^ = 0.083, suggesting that the dysfunction in schizophrenia across PFC regions was dependent on task demands.

**Fig. 5:**
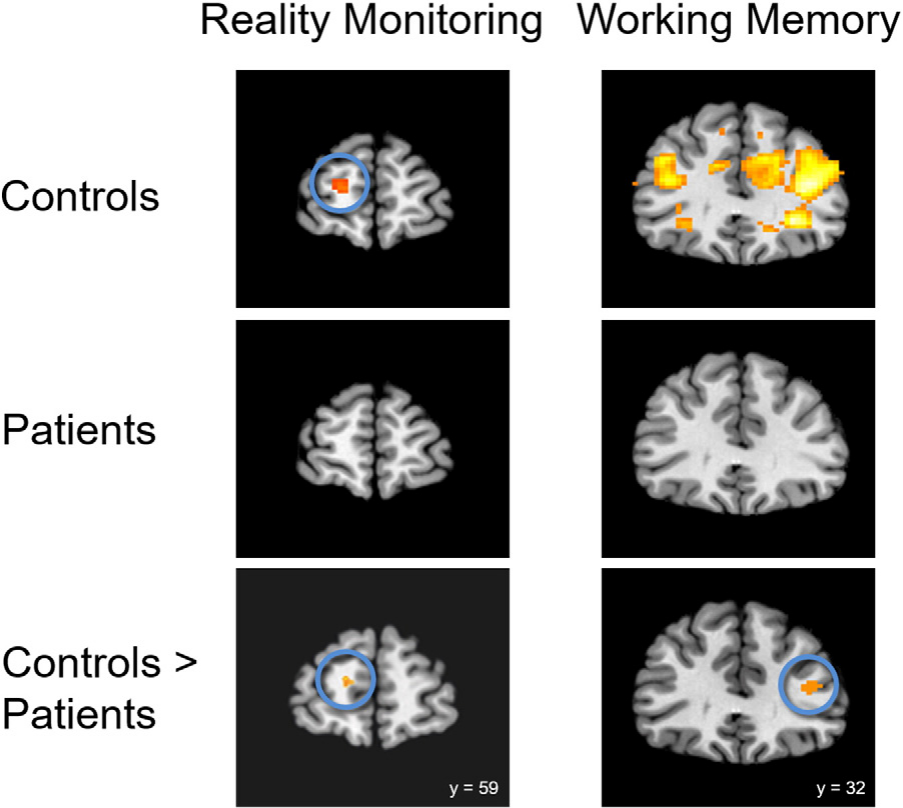
Patients with schizophrenia show reduced medial anterior PFC activity during reality monitoring, and reduced dorsolateral PFC activity during working memory. Left panel: coronal sections taken at z = 8 showing areas of greater activity associated with reality monitoring than perceptual motor baseline. Controls but not patients exhibited significant activity in the left medial anterior PFC (circled, peak: x = −15, y = 59, z = 8). A trend towards significant left hemisphere medial anterior PFC activity was observed in the group contrast of Controls > Patients (circled, peak: x = −15, y = 56, z = 8; p = 0.057). Right panel: coronal sections taken at z = 16 showing areas of greater activity associated with working memory load 5 than load 3. Controls but not patients exhibited significant bilateral activity in the dorsolateral PFC (peak: x = −39, y = 23, z = 28 and x = 39, y = 32, z = 16). Significantly greater dorsolateral PFC activity was observed in the group contrast of Controls > Patients (circled, peak: x = 39, y = 32, z = 14). Activity in all contrasts was significant at voxel-wise height threshold of p < 0.05, small-volume corrected, and is illustrated here for display purposes at p < 0.01, uncorrected.

Exploring the results in more detail using between-group pairwise contrasts (Fig. 5), control participants exhibited significant activity associated with the reality monitoring contrast in the left medial anterior PFC region of interest (peak activity: −15, 59, 8; Z = 2.847) and in the left (peak: −42, 11, 26; Z = 5.124) and right (peak: 45, 32, 18; Z = 3.049) dorsolateral PFC regions of interest, and at a whole-brain corrected voxel-wise height threshold of p < 0.05, in the occipital lobe (peak: 18, −85, −8; Z = 4.645). In patients, significant reality monitoring-related activity was detected only in left dorsolateral PFC (peak: −42, 20, 24; Z = 2.790). A between groups comparison indicated a trend towards a significant reduction in reality monitoring activity in the patients compared with controls in the left medial anterior PFC region of interest only (peak: −15, 56, 8; Z = 2.636, p = 0.057).

Examination of the brain activity associated with the working memory contrast (correct responses at load 5 > load 3) revealed significant activity in the right medial anterior PFC (peak: 21, 53, 6; Z = 3.241), and in the left (peak: −39, 23, 28; Z = 3.890) and right (peak: 39, 32, 16; Z = 4.081) dorsolateral PFC regions of interest in controls, which was not observed in the patients. Neither group exhibited significant activity elsewhere in the brain at a whole-brain corrected voxel-wise height threshold. A between groups comparison revealed that the patients exhibited significantly less load-dependent activity in the right dorsolateral PFC region of interest during working memory relative to controls (peak: 39, 32, 14; Z = 2.674). No regions of significantly greater activity were observed in the patients compared with controls for either the reality monitoring or the working memory contrast.

To confirm that task-specific activity in the two PFC regions was largely unrelated, the next analysis tested the separability of the activity differences that were observed in the between-groups contrasts in the left medial anterior PFC and right dorsolateral PFC regions of interest. A repeated measures ANOVA with the factors of group (patients and controls), task (reality monitoring and working memory), and region (left medial anterior PFC and right dorsolateral PFC), revealed no significant main effects on activity of group, F(1,38) = 1.504, p = 0.228, η_p_^2^ = 0.038, task, F(1,38) < 0.001, p = 0.993, η_p_^2^ = 0, or region, F(1,38) = 0.656, p = 0.423, η_p_^2^ = 0.017. There were also no significant two-way interactions between the factors, F(1,38) < 0.640, p > 0.429, η_p_^2^ < 0.017. However, the three-way interaction between group, task, and region, was significant, F(1,38) = 14.469, p = 0.001, η_p_^2^ = 0.276, consistent with the notion that dissociable patterns of dysfunction in left medial anterior PFC and right dorsolateral PFC underlie the behavioral deficits observed in reality monitoring and working memory, respectively. Supporting this inference, there was no correlation between the percentage signal change for each participant in any of the *a priori* left and right medial anterior and dorsolateral PFC voxels, for the reality monitoring contrast and the working memory contrast, for either patients, (r < 0.200, p > 0.398), or controls, (r < 0.307, p > 0.187).

## 4 Discussion

In this study, patients with schizophrenia exhibited performance deficits during reality monitoring and working memory tasks that were accompanied by distinct activity reductions in the medial anterior and dorsolateral PFC, respectively. This reduced functionality was dissociable by task and region, arguing against either generalized dysfunction across the prefrontal cortex in schizophrenia, or of localized impairment that affected the two tasks similarly. Instead, the results are consistent with separate deficits within prefrontally-mediated brain networks underpinning task performance that manifest as aberrant task-specific cortical activity in different regions of the PFC. Together these results suggest that the reality monitoring impairment that has been reported in schizophrenia has its basis in a pattern of neural dysfunction which is distinct from that underlying the working memory deficits that are typically observed.

The behavioral deficits exhibited by the patients during reality monitoring and working memory were evident across both accuracy and reaction time variables. Notably, there were no significant correlations in performance across tasks for either patients or controls, consistent with the notion that working memory and reality monitoring are supported by largely unrelated processes. Building from these behavioral findings, the within-subjects design of this study enabled comparison of neural activity associated with the two cognitive domains. Only healthy subjects exhibited significant activity in the *a priori* region of left medial anterior PFC during reality monitoring - activity that trended towards being significantly greater than that observed in patient participants. Similarly, controls exhibited significant activity in the *a priori* working memory related regions of bilateral dorsolateral PFC, whereas the patients did not, with activation in the right dorsolateral PFC significantly greater for controls than for patients. The apparent separability of the behavioral impairments in reality monitoring and working memory was supported by the finding of a significant three-way group-by-region-by-task interaction for the analysis of peak signal strength. This interaction, together with an absence of correlation between reality monitoring related and working memory related activity in the medial anterior and dorsolateral PFC, indicates that the neural activity reductions observed in schizophrenia were dissociable and dependent on individual task demands.

The pattern of prefrontal cortical activity observed across the groups during the two tasks enabled a number of alternative explanations of cognitive impairment in schizophrenia to be ruled out. There were no significant main effects of group or task on percentage signal change, and no significant two-way interactions involving the factor of group. The absence of a main effect of task suggests that there were no systematic differences in activity across the two tasks, and the absence of two-way group-by-task interactions indicates no significant difference in overall activity levels between the two groups that were dependent on the task undertaken. Examination of the pattern of percentage signal change across the four regions (left and right medial anterior PFC and dorsolateral PFC) suggested that the significant main effect of region, as well as the interaction between task and region, were driven predominantly by activity in the left dorsolateral PFC which was greater for the reality monitoring task than the working memory task. Notably, no significant activity was observed in the left dorsolateral PFC region in the group comparison of controls compared with patients, suggesting that this region was not associated with the dysfunction that might underlie the reality monitoring and working memory deficits observed in the patients with schizophrenia.

The absence of a main effect of group is particularly notable as it is inconsistent with an explanation of generalized cognitive impairment in schizophrenia (Hill et al., 2004, Hughes et al., 2003, Hyde et al., 1994, Palmer et al., 2010, Palmer et al., 2009), as arising from hypofrontality (Ingvar and Franzen, 1974, Weinberger et al., 1996, Weinberger et al., 1992). Similarly, the lack of significant group-by-region interaction argues against an account in terms of a simple localized PFC neural deficit in the patients. Ruling out these alternative explanations of generalized or regional cognitive dysfunction in schizophrenia, we are left with the implications of the three-way group-by-task-by-region interactions observed in the present data. These findings indicate a dissociation in the neurocognitive impairments related to reality monitoring and working memory task performance in schizophrenia, with the level of activity reduction in the left medial anterior and right dorsolateral PFC varying within patients across the different cognitive domains.

The identification of distinct neurocognitive deficits in schizophrenia is consistent with theoretical models that identify dysconnectivity as a primary pathophysiological mechanism (Andreasen et al., 1999, Bullmore et al., 1997, Friston and Frith, 1995, Stephan et al., 2009, Weinberger et al., 1992). However, the findings suggest no global brain-wide deficit as might be explained by a consistent weakness in network coordination, but instead elements of network-specific dysconnectivity which may vary both within, and between, patients. Such dysconnectivity appears to result in variations in localized cortical activity in patients relative to controls, depending on task requirements. The dysconnectivity explanation is supported by widespread evidence of abnormal structural and functional connectivity in patients with schizophrenia across the brain (van den Heuvel and Fornito, 2014). More specifically, observations of functional dysconnectivity during both working memory and executive function tasks affecting dorsolateral PFC (He et al., 2012, Honey et al., 2002, Kim et al., 2003, Lawrie et al., 2002, Schlösser et al., 2003, Yoon et al., 2008) provide a supportive link between prefrontal activation, task performance and dysconnectivity in underlying cognitive networks.

The task specific dissociation in PFC dysfunction observed in the current study may help to inform an explanation of individual differences in symptomology in schizophrenia. The sample size and homogeneity in symptom data that informed selection of patients for inclusion in the current study precluded exploration of this putative link, but it may be that variation in the experience of hallucinations in schizophrenia can be explained in part by a distinct pattern of dysfunction in the underlying cognitive network subserving reality monitoring, mediated by medial anterior PFC. Consistent with such a possibility, meta-analysis of fMRI data indicates one of the brain regions exhibiting activity during hallucinations to be medial anterior PFC (Zmigrod et al., 2016). Furthermore, morphological variation in the paracingulate sulcus, located within the medial anterior PFC, has been linked both to reality monitoring ability in healthy individuals (Buda et al., 2011) and to the experience of hallucinations in schizophrenia (Garrison et al., 2015), perhaps supporting an explanation of variation in network dysconnectivity as due in part to localized structural differences. Future studies in larger and more heterogeneous patient samples, exploring the links between brain morphology, specific patterns of prefrontal dysfunction and variability in symptoms in schizophrenia, will help to develop and test this account further.

## Financial disclosures

The authors report no biomedical financial interests or potential conflicts of interest.
